# Supplementary material for: Risk Factors Related to Resting Metabolic Rate-Related DNAJC6 Gene Variation in Children with Overweight/Obesity: 3-Year Panel Study
Source: Nutrients. 2024 Dec 23;16(24):4423. doi: 10.3390/nu16244423 (PMC11676649; doi:10.3390/nu16244423)
Supplement: Supplementary file 1 [file nutrients-16-04423-s001.zip › nutrients-3336427-supplementary.pdf]

Supplementary Table S1. Nine SNPs of DNAC6 genes and relative frequencies of major and minor alleles.

No	rs number	primer sequence		Tm	additive
D-I	*rs17127601*	Forward Primer<br>Reverse Primer<br>Genotyping Primer	atggatgaggaagttgagg<br>ttaaaactggaggctggat<br>GCCTCTSCAAACAAATTGAGAAAG	55	betaine
D-II	*rs6697554*	Forward Primer<br>Reverse Primer<br>Genotyping Primer	TGGTTTAGATTGCCTTGAA<br>TTTCATTAACCTGTATCACTGC<br>GTGAGCATTTGCAGTTGTAAC	55	betaine
D-III	*rs1359530*	Forward Primer<br>Reverse Primer<br>Genotyping Primer	attgttcaggcacagtgag<br>gatcgtgctatttgcactc<br>cacagccaacacttatcaagttatc	55	betaine
D-IV	*rs3850826*	Forward Primer<br>Reverse Primer<br>Genotyping Primer	aacagcgtgtaagttgcag<br>TCCACACCAAAGATTCTGA<br>cttttattctggtagtccatgcgac	5	betaine
D-V	*rs6588132*	Forward Primer<br>Reverse Primer<br>Genotyping Primer	CTTTGACTCACCCCATCTT<br>ATGCTACAAGGCAAGGACT<br>CAGAAGCCATGGTGGAGATTTGAATG	55	betaine
D-VI	*rs10789182*	Forward Primer<br>Reverse Primer<br>Genotyping Primer	AGCTAAGGACTCAGGGTGA<br>GGACTTGAGCTCCTGTGAT<br>catattcctgcttcttagtagtttt	55	betaine
D-VII	*rs1334880*	Forward Primer<br>Reverse Primer<br>Genotyping Primer	TCCACAACCACTGCTACTT<br>AGCAGAGGACTGATGGTCT<br>ggagctaagagaagacaatcttggt	55	betaine
D-VIII	*rs735489*	Forward Primer<br>Reverse Primer<br>Genotyping Primer	gagcttgagaaaacgcttc<br>AATTACCACCCTGGAACAG<br>CTCTTTGCAGTTTTCTTTAAG	55	betaine
D-IX	*rs1334881*	Forward Primer<br>Reverse Primer<br>Genotyping Primer	gaaggaggcctagaggagt<br>AGGAAGGAACTCGTGTCAT<br>gcttttaagcagaaaaaccaa	55	betaine
